# QTLs associated with phenolic acid accumulation and antioxidant activity in tropical maize

**DOI:** 10.3389/fpls.2026.1876404

**Published:** 2026-08-18

**Authors:** Silverio García-Lara, Tzitziki González-Rodríguez, David J. Bergvinson

**Affiliations:** 1 Escuela de Ingeniería y Ciencias, Tecnológico de Monterrey, Monterrey, NL, Mexico; 2 Agricultural Development, Gates Foundation, Seattle, WA, United States

**Keywords:** antioxidant capacity, phenolic acids, phytochemicals, QTLs, tropical maize

## Abstract

Maize represents a significant source of phytochemicals, with phenolic acids standing out as one of the most extensively studied functional compound families. These bioactive molecules have gained attention for their potent antioxidant properties and potential contributions to human health improvement. To evaluate the segregation of phenolic compounds in maize and its genetic basis, this study was conducted to identify quantitative trait loci (QTLs) associated with major phenolic compounds and their antioxidant capacity. The mapping population comprised 100 recombinant inbred lines (RILs) derived from the cross between P84 and Kilima. Twelve traits were analyzed: free and cell wall-bound antioxidant capacity, total phenolic content, and contents of *p*-coumaric acid, ferulic acid, three isomers of di-ferulic acid, and three isomers of tri-ferulic acid. The RILs exhibited substantial diversity in phenolic compound profiles. In total, 19 QTLs were identified for nine traits, with the number of associated regions ranging from 1 to 5 and explaining between 2.95% and 37.48% of the phenotypic variation. This research provides substantial evidence for the co-localization of major QTLs for principal phenolic acids in maize with genomic regions harboring genes putatively related to their biosynthesis and biotic resistance. This is the first study to report QTLs associated with triferulic acids in maize. The identified regions co-localizing with biotic stress resistance genes represent targets for marker-assisted selection toward the improvement of phenolic acid accumulation in maize breeding programs.

## 1 Introduction

Maize (*Zea mays* L.) is one of the most widely cultivated cereal crops worldwide, with global production reaching approximately 1,236 million tons in 2024, and serving as a staple for both food and feed in developing regions (OECD and FAO, 2023). However, ensuring sufficient maize production is no longer adequate to meet the demands of a growing population. Climate change, shifting dietary patterns, and the increasing prevalence of diet-related diseases have shifted attention toward improving not only yield, but also the nutritional and functional quality of crops (Zavala-López et al., 2020). In this context, enhancing maize with bioactive secondary metabolites has emerged as a promising strategy to simultaneously address human health and agricultural sustainability (Tiessen-Favier et al., 2022).

Among these metabolites, phenolic compounds are widely distributed across maize germplasm and represent a key class of functional phytochemicals (Adom and Liu, 2002; Pérez Ruiz et al., 2024). Phenolic acids, particularly hydroxycinnamic acid derivatives, have attracted attention for their antioxidant capacity, anti-inflammatory activity, and association with a reduced risk of chronic diseases (Domínguez-Hernández et al., 2022; González-Rodríguez and García-Lara, 2024). In maize kernels, phenolic acids are present in both soluble and cell wall-bound forms. While the soluble fraction is concentrated in the outer grain layers, the bound fraction represents the majority of total phenolics and consists primarily of ferulic acid and related compounds esterified to hemicellulose (Ndolo and Beta, 2018).

Beyond their nutritional relevance, bound phenolic acids play a critical role in plant defense. Ferulic acid has been negatively associated with susceptibility to storage pests such as *Sitophilus zeamais*, with higher concentrations contributing to increased resistance. More structurally complex derivatives, including diferulic acids (DiFA) and triferulic acids (TriFA), further reinforce this defense by forming covalent cross-links within the cell wall, enhancing its rigidity and limiting pest penetration (Bily et al., 2003; García‐Lara et al., 2004; Santiago et al., 2013). While DiFA has also been linked to resistance against fungal pathogens, TriFA remains poorly understood, despite evidence of its presence and structural characterization in maize bran (Ayala-Soto et al., 2014; Hussain et al., 2021).

The phenolic composition of maize exhibits substantial variation across germplasm, including landraces, inbred lines, and diverse geographic accessions (Feregrino-Pérez et al., 2024; Fuentes-Cardenas et al., 2022; Gong et al., 2025; González-Muñoz et al., 2013; Libron et al., 2021; Zhang et al., 2025). This variability has enabled the identification of high-phenolic sources and the development of improved populations through recurrent selection (García-Lara and Bergvinson, 2014). Although previous studies have mapped QTLs associated with phenolic acids such as ferulic acid and DiFA in early-generation populations (Castro-Álvarez et al., 2015), the genetic architecture underlying these traits remains incompletely resolved. In particular, the genomic regions controlling structurally complex phenolics, such as TriFA, have not been reported to date.

Phenolic acid accumulation is a quantitative trait controlled by multiple loci, making QTL analysis a suitable approach for its genetic dissection. Advances in molecular marker technologies and linkage mapping have facilitated the identification of genomic regions associated with complex traits in maize, extending the potential of QTL analysis to complex quality traits such as phytochemical composition (González-Rodríguez et al., 2025; Hasan et al., 2021; He et al., 2025). However, the use of advanced recombinant inbred line (RIL) populations, which provide higher recombination frequency and improved mapping resolution, remains limited for phenolic traits.

Therefore, considering the importance of maize kernel phytochemical composition for both human nutrition and crop resilience, the present study aimed to characterize the genetic basis of phenolic acids in an advanced F_6_ RIL population derived from a cross between a high-phenolic, pest-resistant tropical line (P84) and an adapted tropical variety (Kilima). Specifically, we aimed to: (1) identify QTLs associated with phenolic acid composition and antioxidant capacity, (2) estimate their genetic effects and contribution to phenotypic variance, and (3) evaluate the stability of these loci across generations by comparison with previously reported QTLs and identify priority targets for marker-assisted breeding.

## 2 Materials and methods

### 2.1 Mapping population

High phenolic acid maize was developed to the RIL level using parent lines derived from Population 84 (P84) and Kilima. P84 was selected for its resistance to storage pests (Kumar, 2002) and high levels of phenolic acids (García-Lara and Bergvinson, 2014), while Kilima was chosen for its adaptability to tropical environments. The mapping population was developed by selfing after crossing the lines (P84 C1 and Kilima ST94A) through six successive growing environments until RIL F_6_ was achieved under subtropical conditions (Tlaltizapán, Morelos, México, 18°41′N lat., 939 masl). The population was developed between 2006 and 2010 using a randomized complete block design. At the F_4_ level of recombination, 397 families were evaluated for insect resistance following the extreme analysis procedure (Mansur et al., 1993). From these lines, 100 RILs were selected for QTL mapping based on phenotypic extremes for phenolic acid content. To obtain sufficient grain for phytochemical analyses, seed of the selected F_6_ RILs was increased during the 2022 and 2023 growing seasons at CIMMYT’s Agua Fría Experimental Station, Puebla, Mexico (19° N, 60 masl). Ears from each family were harvested, dried, shelled, and stored at 4°C until further processing.

### 2.2 Molecular marker mapping

The map assembly based on molecular markers was developed as detailed by (Castro-Álvarez et al., 2015). In summary, DNA was extracted from the leaf tissue of parental lines and RIL families using the CTAB method. A linkage map was created using three hundred thirty-eight simple sequence repeat (SSR) markers that ensure uniform and complete coverage of the entire maize genome. HyperMap data was used to capture molecular marker data, and segregation was tested for deviations from the expected Mendelian ratios in an RIL population (1:1) using a standard χ² test. The linkage map was constructed with Haldane’s mapping function using the MAPMAKER software package (Lander et al., 1987). One hundred RIL families were selected based on polymorphism for SSR mapping and subsequent QTL analysis.

### 2.3 Phenolics compounds analysis

#### 2.3.1 Extraction

Kernels from the parental lines and RIL families were dissected to obtain the pericarp fraction following the method described by (García‐Lara et al., 2004). Pericarps were milled using a ball mill (Retsch Mixer Mill MM 400, Haan, Germany), and the ground samples were used for the extraction as previously detailed in (Zavala-López and García-Lara 2017). Briefly, soluble phenolics were extracted in triplicate from each sample (50 mg) using 80% methanol. The remaining pellet was subjected to alkaline hydrolysis for 1 hour, followed by neutralization. Lipids were removed through two hexane washes, and cell-wall-bound phenolics were recovered via three ethyl-acetate washes. The bound phenolic extracts were dried under gaseous nitrogen and dissolved in 50% methanol. All extracts were stored at -20 °C until further analysis.

#### 2.3.2 Quantification of total content

Total phenolics were determined by the Folin-Ciocalteu method following the protocol described in (Urias-Peraldí et al., 2013). Results were expressed as μg of gallic acid equivalents per 100 g dry weight (mg GAE/100 g dw).

#### 2.3.3 Profiling by HPLC

Phenolic compounds were analyzed using a high-performance liquid chromatography (HPLC) system coupled with a photodiode array detector (PDA) (Agilent 1100, Santa Clara, CA), as previously described by (Zavala-López and García-Lara, 2017). Compounds were separated on a Zorbax SB-Aq reverse phase column (4.6 mm × 150 mm, 3.5 μm) using a linear gradient of acidified water (pH 2) and acetonitrile (CAS: 75-05-8, BDH, West Chester, PA, USA) at a flow rate of 0.6 mL/min and 25 °C. Data were processed using ChemStation software (Agilent Technologies, 1990–2003). Results were expressed as mg per g dry weight (mg/g dw).

### 2.4 Measurement of antioxidant capacity

Antioxidant capacity was measured using the oxygen radical absorbance capacity (ORAC) assay according to (Zavala-López & García-Lara, 2017) with minor modifications. The assay utilized Trolox as standard, fluorescein as the probe, and 2,2´-azobis(2-amidinopropane) dihydrochloride (AAPH) as the peroxyl radical generator. Measurements were performed using a microplate reader (Synergy™ HT Multi-Detection, BioTek, Inc., Winooski, VT, USA). Results were expressed as Trolox equivalents (µmol of Trolox/100g dw).

### 2.5 Phenotypic data analysis

Grain phenolic compounds were analyzed using analysis of variance (ANOVA) with Statistix v.7 (Analytical Software, Tallahassee, FL, USA). Estimates of genotypic variance were calculated by equating mean squares to their expected values, under the assumption that the data set followed a normal distribution. Gaussian distribution and transgressive segregation were evaluated within the RIL population. Pairwise phenotypic correlations among phytochemical traits were estimated using Pearson’s correlation coefficient, and the statistical significance of the correlation coefficients was calculated using Statistix v.7. The resulting correlation matrix was visualized as a heatmap using the *corrplot* package in R Studio (R Core Team, 2025). Since phenotypic data were collected in a single environment, heritability, QTL × environment (QTL × E) interaction effects, and the proportion of genotypic variance explained by individual QTL were not estimated.

### 2.6 QTL mapping analysis

Phenolic acids and antioxidant activity were used as phenotypic traits in the QTL analysis with 100 representative RILs from the 397-line population, employing composite interval mapping (CIM) within a single environment (Jansen and Stam, 1994; Jiang and Zeng, 1995). Following the methodology described by (Castro-Álvarez et al., 2015), three sequential models (III, II, and I) were applied for QTL detection. Model III, corresponding to o simple interval mapping, was used for the selection of marker cofactors. Model II was subsequently fitted using the selected markers as cofactors, provided they were unlinked to the genomic region of interest. Finally, Model I was used to confirm the QTL detected in Model II. Model I included all selected cofactors together with markers flanking the target interval using window sizes of 20 and 30 cM. A QTL was declared when the likelihood ratio (LR) exceeded the selected threshold in Model II and the corresponding peak was also significant in Model III using both window sizes (Castro-Álvarez et al., 2015). The thresholds used for QTL detection for one environment were a LR of 13.81 (equivalent to LOD = 3.0), LR = 11.51 (LOD = 2.5), and LR = 9.21 (LOD = 2.0). These critical values are equivalent to a significance level of α = 0.00317, α = 0.00925, and α = 0.0266, respectively, in a χ2 distribution with df = 2 in a RIL population. The phenotypic variance explained by QTL was obtained by the coefficient of determination (R²). Finally, co-localization of QTLs from the F_2:3_ to F_6_ was declared when QTL positions overlapped within the same chromosomal bin (Castro-Álvarez et al., 2015). The bin was set for each QTL as the position of the closest marker according to the Maize Genetics and Genomics Database [MaizeGDB (Portwood et al., 2019)].

## 3 Results

### 3.1 Phenotypic variation in phenolic acids and antioxidant activity

The means values of the parental lines P84 (high phenolic acid content) and Kilima (low phenolic acid content) differed significantly (*p* < 0.05) for most phenolic acids measured in the evaluated environment. For total phenolic acids, including both free (FPA) and bound (BPA) fractions, as well as their associated antioxidant activity (free antioxidant capacity, FAox; and bound antioxidant capacity, BAox), also showed significant differences between parental lines (*p* < 0.05). Bound phenolic acids were 34-fold more abundant than free phenolic acids, and antioxidant activity followed the same pattern, with BAox being 20-fold higher than FAox (**Table 1**).

**Table 1:** Means and estimates of variance components for total phenolic content and antioxidant activity of maize kernel, over one season for parent with high and low phenolic content in RILs (F_6_) families from the cross P84 × Kilima.

Means	Phenolic acids (mg GAE/100 g dw)^a^		Antioxidant activity (µmol of trolox/100 g dw)	
	Total free	Total bound	Total free	Total bound
P84^b^	71.41 ± 3.34	2440.11 ± 10.41	19.68 ± 3.55	117.61 ± 10.02
Kilima^c^	44.38 ± 4.30	1501.64 ± 10.29	2.13 ± 2.94	44.20 ± 4.02
PM ^d^	57.89 ± 4.33	1980.12 ± 11.77	10.74 ± 3.01	80.50 ± 3.21
F_6_ ^e^	58.62 ± 6.98	1226.35 ± 24.91	11.90 ± 4.21	82.58 ± 5.23
Range	41.26-80.65	1147.52-2289.47	1.45-13.53	40.18-120.69
σ^2g^	7.137*	116.932**	0.120**	9.569*

^a^ GAE, gallic acid equivalents.

^b^ Parental line with high phenolic acid content, n = 10

^c^ Parental line with low phenolic acid content, n = 10

^d^ PM = mean of P84 and Kilima, n = 14

^e^ F_6_ = mean of F_6_ lines, n = 120

Significance levels: *p < 0.05; **p < 0.01.

Phenotypic distributions among RIL families were approximately Gaussian, with some traits extending beyond the parental ranges, indicating transgressive segregation (**Supplementary Figure 1**). Free phenolic acids ranged from 41.26 to 80.65 mg GAE/100 g dw, exceeding the parental range of 44.38–71.41 mg GAE/100 g dw, whereas bound phenolic acids ranged from 1147.52 to 2289.47 mg GAE/100 g dw compared with a parental range of 1501.64–2440.11 mg GAE/100 g dw. Free antioxidant capacity ranged from 1.45 to 13.53 µmol Trolox/100 g dw relative to 2.13–19.68 µmol Trolox/100 g dw in the parental lines, while bound antioxidant capacity ranged from 40.18 to 120.69 µmol Trolox/100 g dw compared with 44.20–117.61 µmol Trolox/100 g dw (**Table 1**).

### 3.2 Composition of cell wall bound phenolic acids

A detailed analysis of bound phenolic acids (BPA) revealed the presence of simple, di-, and tri-ferulic acids (**Table 2**). The simple phenolics acids identified included *p*-coumaric acid and trans-ferulic acid, as well as three isomers of diferulic acid (DiFA): 5,5’-DiFA, 8,5’-DiFA, and 8-O-4’-DiFA. Triferulic acids (TriFA) were also detected, including 5-5’, 8-O-4(H_2_O)-TriFA, 8-O-4’, 8-O-4’-TriFA, and 8-8’, 8-O-4’-TriFA. Trans-ferulic acid represented over 80% of total phenolic acids in RIL families, whereas DiFA and *p*-coumaric acid accounted for 9% and for 5%, respectively.

**Table 2:** Means and estimates of variance components for phytochemical parameters of maize kernel, over one season for the parental lines and population used in this study.

Means	Phenolic acids (mg/g dw)^a^							
	Simple Phenolics		Diferulic acids - DiFA			Triferulic acids - TriFA		
	*p*-CA	*trans*-FA	5,5’-DiFA	8,5’-DiFA	8-O-4’-DiFA	5-5’, 8-O-4(H_2_O)-TriFA	8-O-4’, 8-O-4’, TriFA	8-8’, 8-O-4’, TriFA
P84^b^	2.71 ± 0.13	38.11 ± 0.44	2.80 ± 0.04	0.61 ± 0.02	0.93 ± 0.02	0.53 ± 0.01	0.30 ± 0.01	0.81 ± 0.01
Kilima^c^	1.40 ± 0.10	18.14 ± 0.38	1.35 ± 0.03	0.20 ± 0.02	0.62 ± 0.03	0.48 ± 0.03	0.27 ± 0.01	0.77 ± 0.01
PM ^d^	2.05 ± 0.12	28.12 ± 1.56	2.07 ± 0.03	0.40 ± 0.02	0.77 ± 0.02	0.50 ± 0.03	0.28 ± 0.01	0.79 ± 0.02
F_6_ ^e^	2.34 ± 0.70	26.35 ± 4.09	1.90 ± 0.43	0.32 ± 0.12	0.80 ± 0.14	0.80 ± 0.29	0.41 ± 0.12	0.94 ± 0.14
Range	1.46-3.98	17.52-39.19	1.76-3.53	0.18-0.69	0.58-1.28	0.78-1.36	0.30-0.69	0.61-1.21
σ^2g^	0.143**	6.392*	0.020**	0.001	0.004*	0.002*	0.001	0.003*

^a^ *p*-CA, coumaric acid; FA, ferulic acid; DiFAs and TriFAs were quantified as equivalent of ferulic acid.

^b^ Parent with high phenolic acid content, n = 10

^c^ Parent with low phenolic acid content, n = 10

^d^ PM = mean of P84 and Kilima, n = 14

^e^ F_6_ = mean of F_6_ lines, n = 120

Significance levels: *p < 0.05; **p < 0.01.

Among diferulic acid isomers, 5,5’-DiFA was the most abundant, while for triferulic acids was 8-8’-, 8-O-4-TriFA isomer. Mean values of F_6_ RIL families were compared with the mid-parent mean (PM). The means of F_6_ RIL families for *p*-coumaric acid, 8-O-4’-DiFA, and all three TriFA isomers were significantly (*p* < 0.05) greater than the PM, with some families exceeding the parental ranges. In contrast, trans-ferulic acid, 5,5’-DiFA and 8,5’-DiFA showed F_6_ means below the PM (**Table 2**). For example, trans-ferulic acid ranged from 17.52 to 39.19 mg/g dw compared with a parental range of 18.14-38.11 mg/g dw, whereas 5,5’-DiFA varied from 1.76 to 3.53 mg/g dw relative to 1.35-2.80 mg/g dw in the parental lines.

**Figure 1:**
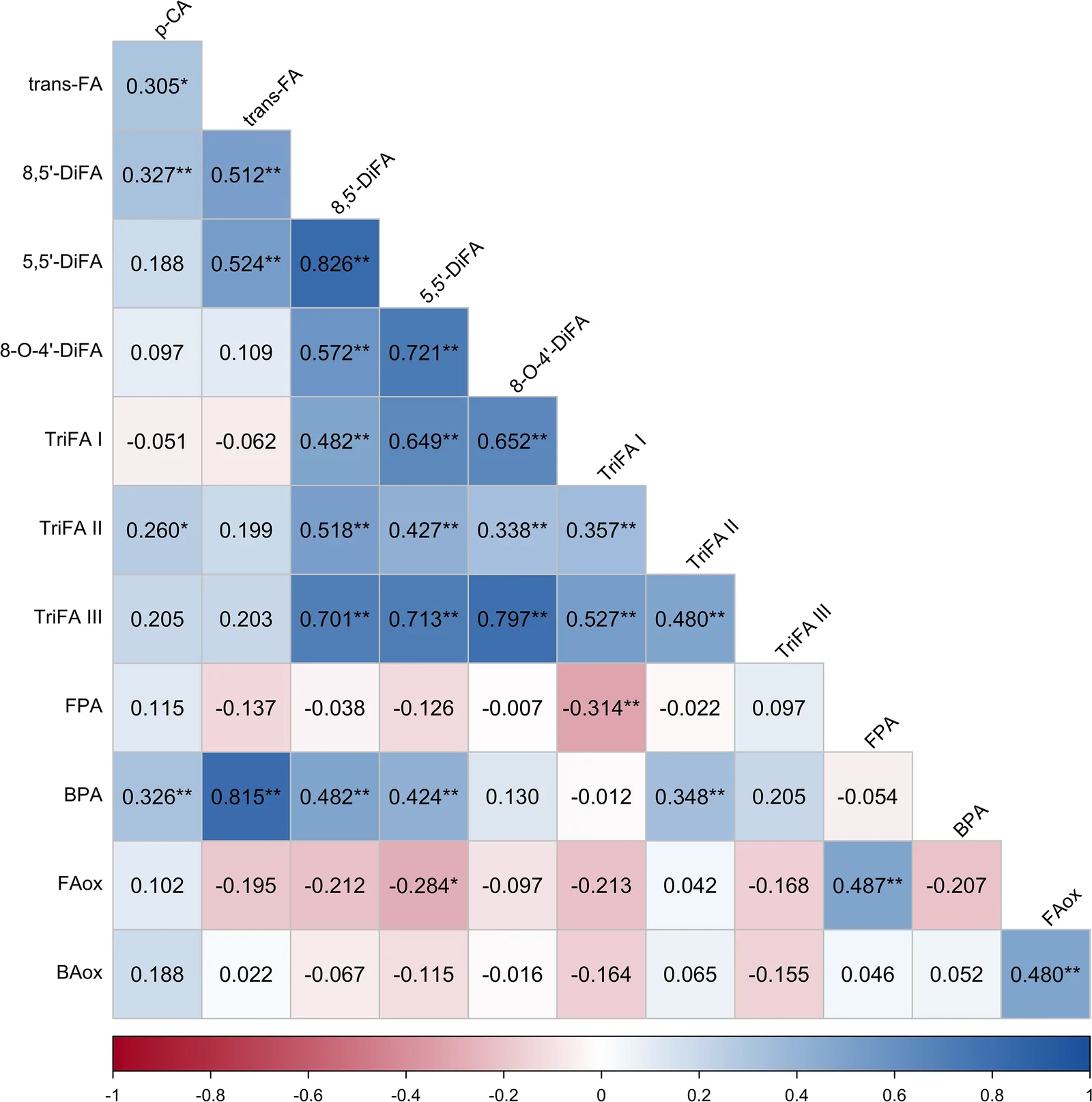
Phenotypic correlations among phytochemical parameters of maize kernels, over one season in RILs (F_6_) families derived from the cross between P84 × Kilima. Blue and red indicate positive and negative correlation, respectively. *p*-CA, *p*-coumaric acid; FA, ferulic acid; DiFA, diferulic acids; TriFA, triferulic acids; FPA, free phenolic acids; BPA, bound phenolic acids; FAox, free antioxidant capacity; BAox, bound antioxidant capacity; TriFA I, 5-5’, 8-O-4’ (H_2_O)-TriFA; TriFA II: 8-O-4’,8-O-4’ TriFA; TriFA III: 8-8’,8-O-4’ TriFA. Correlation significance levels: *p < 0.05; **p < 0.01.

Correlation analysis revealed significant associations (*p* < 0.05) among phenolic compounds and between selected antioxidant capacity measurements and their corresponding phenolic fractions (**Figure 1**). Strong positive correlations were observed between DiFA and TriFA isomers (*r* > 0.700; *p* = 0.01), and between trans-ferulic acid and bound phenolic acids (*r* = 0.815; *p* = 0.01). DiFA isomers were also highly intercorrelated (*r* = 0.826; *p* = 0.01). Regarding antioxidant activities, free antioxidant capacity (FAox) was significantly correlated with total free phenolic acids (FPA) (*r* = 0.487; *p* = 0.001); FAox and bound antioxidant capacity (BAox) were also significantly correlated with each other (*r* = 0.480; *p* = 0.01). Notably, the correlation between BAox and total BPA was not statistically significant (*r* = 0.052), suggesting that BAox may be influenced by compounds beyond those quantified in this study (**Figure 1**).

### 3.3 QTLs for phenolic acids and antioxidant activity

**Figure 2:**
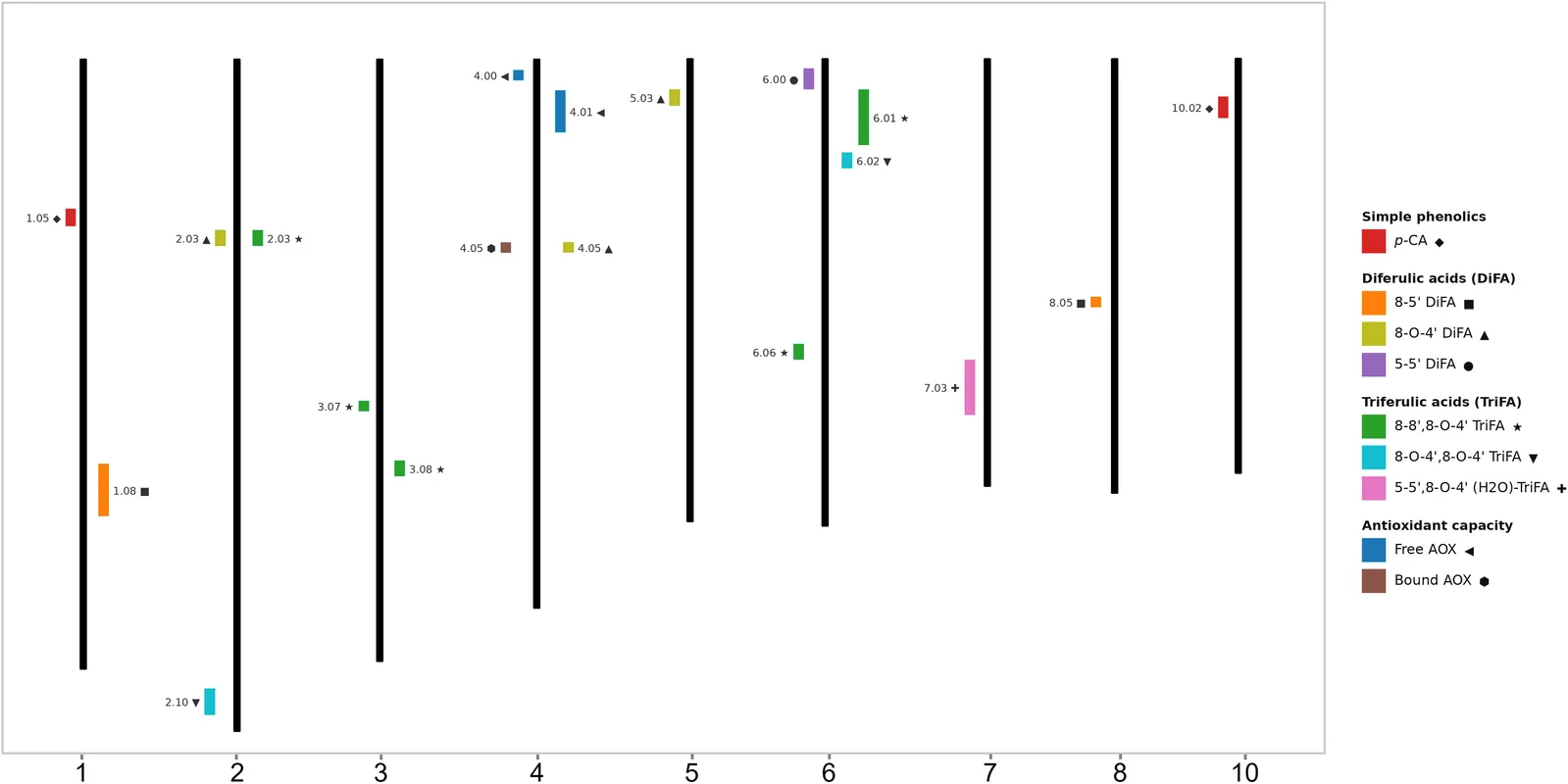
Genetic map of QTLs associated with phytochemical composition. Combined SSR linkage map based on 100 F_6_ families derived from the cross P84 × Kilima. Maize chromosomes are represented with black bars. QTL positions for phytochemical traits are shown in different colors. Each trait is additionally identified by a unique symbol to ensure that the figure remains interpretable regardless of color. *p*-CA, *p*-coumaric acid; FA, ferulic acid; DiFA, diferulic acids; TriFA, triferulic acids; FPA, free phenolic acids; BPA, bound phenolic acids; FAox, free antioxidant capacity; BAox, bound antioxidant capacity.

QTL analysis was performed using an SSR linkage molecular map of subtropical maize previously reported (Castro-Álvarez et al., 2015), consistent with MaizeGDB (Portwood et al., 2019). A total of 19 genetic regions were identified across nine traits and were located on nine chromosomes of maize (**Figure 2**; **Table 3**).

**Table 3:** QTL regions identified for phenolic acid compounds and antioxidant capacity in 100 F_6_ RIL families of tropical maize, detected by composite interval mapping.

Trait^a^	Bin^b^	Marker interval	QTL^c^		
			LOD	LR	R^2^ (%)
Free antioxidant capacity	4.00	*umc2278-umc1008*	3.17	14.60	17.77
	4.01	*umc1228-bnlg1241*	2.89	13.31	11.71
					**18.07**
Bound antioxidant capacity	4.05	*bnlg490-bnlg1265*	2.81	12.93	2.95
					**2.95**
*p*-coumaric acid	1.05	*bnlg1884-umc2025*	2.85	13.14	11.67
	10.02	*umc2018-mmc0501*	4.66	21.46	20.65
					**34.07**
5-5’-DiFA	6.00	*phi126-bnlg1883*	2.18	10.05	9.45
					**9.45**
8-5’-DiFA	1.08	*phi037-dup128*	2.25	10.34	2.99
	8.05	*umc2367-umc1309*	3.26	15.02	8.70
					**15.51**
8-O-4’-DiFA	2.03	*bnlg1064-umc1185*	3.07	14.14	14.31
	4.05	*bnlg490-bnlg1265*	3.00	13.82	10.72
	5.03	*bnlg1046-phi113*	2.05	9.45	5.30
					**28.05**
5-5’, 8-O-4’ (H_2_O)-TriFA	7.03	*bnlg1070-phi114*	2.14	9.87	10.36
					**10.36**
8-O-4’, 8-O-4’-TriFA	2.10	*umc2184-phi101049*	2.58	11.89	9.35
	6.02	*umc1006-umc1723*	2.05	9.46	8.40
					**19.07**
8-8’, 8-O-4’-TriFA	2.03	*bnlg1064-umc1185*	2.30	10.6	10.53
	3.07	*umc1644-bnlg1931*	2.83	13.04	5.26
	3.08	*bnlg1779-bnlg1108*	3.21	14.79	10.90
	6.01	*bnlg1883-phi077*	3.48	16.02	12.52
	6.06	*bnlg345-umc1859*	2.15	9.90	8.99
					**37.48**

^a^ DiFA, diferulic acids; TriFA, triferulic acids.

^b^ Chromosome location of each QTL is based on the bins concept.

^c^ Likelihood ratio was estimated under model II using markers as cofactors. Phenotypic variances explained by each QTL and all (bold) QTL affecting the trait.

For total phenolic acids (both free and bound), no significant QTL regions were found. In contrast, two QTL regions related to the antioxidant capacity of free phenolic acids (FAox) were identified on chromosome 4 (bins 4.0 and 4.01), along with one QTL for BAox in bin 4.05, explaining 18.07% and 2.95% of the phenotypic variance (σ²p), respectively. Individually, the FAox QTL in bin 4.00 accounts for 17.77% of σ²p, while the FAox QTL in bin 4.01 accounts for 11.71% of σ²p. No genes putatively associated with phenolic biosynthesis were identified in these bins; however, bin 4.01 contains a gene conferring resistance to *Puccinia sorghi* (**Table 4**).

**Table 4:** Genomic bins containing QTLs identified in this study, with co-localizing genes and QTLs retrieved from MaizeGDB (http://www.maizegdb.org/).

Chr.Bin	Associated trait	Putatively associated gene	Associated QTL
1.05	*p*-coumaric acid	*Exserohilum turcicum* response (*ht4*)	Response to *Cercospora zeae-maydis*
		Soft endosperm (*sen3*)	Maysin content
			300-kernel weight
1.08	8-5’-DiFA	Soft endosperm *(sen3*)	300-kernel weight
			European corn borer response
2.03	8-O-4’-DiFA and	Peroxidase gene (*px14*)	European corn borer response
	8-8', 8-O-4'-TriFA	Soft endosperm (*sen5*)	1000-kernel weight
2.10	8-O-4', 8-O-4'-TriFA	Soft endosperm (*sen5*)	
		Peroxidase (*px1*)	
3.07	8-8', 8-O-4'-TriFA	Soft endosperm (*sen1*)	300-kernel weight
			Southwestern corn borer response
3.08	8-8', 8-O-4'-TriFA	Soft endosperm (*sen1*)	1000-kernel weight
			Southwestern corn borer response
4.01	Free antioxidant capacity	*Puccinia sorghi* resistance (*rp4*)	
4.05	Bound antioxidant capacity	Brittle endosperm (*bt2*)	Response to *Cercospora zeae-maydis*
	8-O-4’-DiFA	Phenylalanine ammonia lyase (*pal3*)	300-kernel weight
5.03	8-O-4’-DiFA	Soft endosperm (*sen6*)	300-kernel weight
			Response to *Cercospora zeae-maydis*
6.00	5-5’-DiFA	Resistance to *Bipolaris maydis* (*rhm2*)	Maysin content
6.01	8-8', 8-O-4'-TriFA	Resistance to *Bipolaris maydis* (*rhm2*)	Response to corn earworm
		Reaction to *X. oryzae* (*rxo1*)	Maysin content
			Response to *Cercospora zeae-maydis*
6.02	8-O-4', 8-O-4'-TriFA	Maize insect resistance (*mir1*)	Response to *Cercospora zeae-maydis*
		Maize insect resistance (*mir2*)	
6.06	8-8', 8-O-4'-TriFA		300-kernel weight
			Maysin content
7.03	5-5', 8-O-4' (H_2_O)-TriFA	Soft endosperm (*sen2*)	300-kernel weight
			Sugarcane borer response
			European corn borer response
8.05-8.06	8-5'-DiFA	*Exserohilum turcicum* response (*ht2/htn1*)	Maysin content
			Response to *Cercospora zeina*
			European corn borer response
			300-kernel weight
10.02	*p*-coumaric acid	Shikimate dehydrogenase (*sad1*)	

DiFA, diferulic acids; TriFA, triferulic acids.

For *p*-coumaric acid (*p*-CA), two QTL regions were detected in bins 1.05 and 10.02, with the latter explaining the highest phenotypic variation of 20.65% (**Table 3**). The wide diversification of *p*-CA content observed across maize grain fractions and products makes it challenging to establish a direct relationship between specific genomic regions and compound accumulation, which may explain why *p*-CA was the only simple phenolic acid for which significant QTLs were identified. According to MaizeGDB, bins 1.05 and 10.02 are associated with characteristics related to phenolic content, which is expected due to the traits analyzed (**Table 4**). In bin 1.05, there is a *Exserohilum turcicum* response gene (*ht4*), involved in resistance to the foliar infection that causes yield loss through reduced photosynthetic leaf area (Ogliari et al., 2005). Also present in bin 1.05 is a soft endosperm gene (*sen3*), a biophysical parameter related to grain hardness and involved in resistance or susceptibility of maize against postharvest pests. Also, QTLs for maysin content and response to *Cercospora zeae-maydis* are previously reported in this bin (**Table 4**). Bin 10.02 contains a shikimate dehydrogenase gene (*sad1*), an enzyme of the shikimate pathway that precedes the formation of phenylalanine, the aromatic amino acid representing the beginning of phenolic acids synthesis.

For diferulic acids (DiFA), six QTL regions were identified across chromosomes 1, 2, 4, 5, 6, 7 and 8 (**Figure 2**; **Table 3**). The QTL for 8-5’-DiFA on bin 8.05 explained 8.7% of σ²p, while the QTL for 5-5’-DiFA on bin 6.00 explained 9.45% of σ²p. These regions co-localized with QTLs reported in previous studies (García-Lara et al., 2010).

For triferulic acids (TriFA), eight QTL regions were detected. The QTL for 5-5’, 8-O-4’ (H_2_O)-TriFA on chromosome 7 (bin 7.03) explained 10.36% of σ²p, while the QTL for 8-O-4’, 8-O-4’-TriFA on bin 2.10 explained 9.35% of σ²p. The highest phenotypic variation was explained by QTLs for 8-8’, 8-O-4’-TriFA (37.48%), with the QTL on bin 6.01 alone contributing 12.52%. Notably, this is the first study to report QTLs associated with TriFA in maize. Some QTL regions co-localized with genomic regions harboring genes putatively associated with biotic stress resistance, including soft endosperm genes, peroxidase genes, and QTLs for maysin content and insect resistance (**Table 4**).

## 4 Discussion

The phenolic acid content and antioxidant activity observed in the parental lines and F_6_ families are consistent with previous reports in native and inbred maize germplasm (García-Lara et al., 2010; Zavala-López et al., 2020). The significant differences observed between the parental lines highlight the genetic variability underlying phenolic compound accumulation. Transgressive segregation was evident across multiple traits, with RIL families exceeding both parental extremes (**Table 1**; **Supplementary Figure 1**), a pattern consistent with the action of complementary alleles from each parent and indicative of substantial additive genetic variance. The higher abundance and bioactivity of bound phenolic acids compared to free phenolic acids align with previous studies, emphasizing their role in plant defense mechanisms (Duncan et al., 2019; García-Lara et al., 2010; Soujanya et al., 2021; Zhang et al., 2025).

Ferulic acid has been largely associated with kernel insect resistance. Early studies established a negative correlation between trans-ferulic acid content in landraces and susceptibility parameters towards maize weevil (Arnason et al., 1994). In a later study comparing phenotypic characteristics of maize kernel in susceptible varieties and resistant maize landraces, trans-ferulic acid was one of the most important phenolic acids explaining phenotypic variance in maize weevil resistance (García‐Lara et al., 2004). The concentration of FA in the pericarp of our high-phenolic parent (38.11 ± 0.44 mg/g dw) stands out as exceptionally high when compared to recent screenings of maize diversity. For instance (Gong et al., 2025), reported a maximum total ferulic acid content of 6.62 mg/g among 233 inbred lines. While their study evaluated whole kernels, the high concentration observed in the pericarp of P84 parent, and its consistency in the F_6_ offspring (up to 39.19 mg/g), confirms that these lines are a superior source for increasing ferulic acid in maize. The presence of diferulic and triferulic acids further supports their role in fortifying the pericarp cell wall, increasing physical strength and kernel hardness and resistance to biotic stress (Bily et al., 2003; García‐Lara et al., 2004). Moreover, 8-O-4-DiFA has been negatively correlated with maize kernel hardness (Chiremba et al., 2012). The strong positive correlations observed among DiFA and TriFA isomers (**Figure 1**) are consistent with the coordinated accumulation of structurally related hydroxycinnamates and provide additional context for the co-localization of several QTL identified in this study. Because these relationships are based on phenotypic correlations, they should not be interpreted as evidence of shared genetic regulation. From a breeding perspective, FAox showed a significant negative correlation with 5,5’-DiFA (r = -0.284, *p* < 0.05), contrasting with its positive correlation with FPA (r = 0.487, *p* < 0.001), whereas no significant correlation was observed between FAox and any TriFA isomer (r = -0.213 to 0.042). This divergent pattern suggests that simultaneous selection for FAox, FPA, and 5,5’-DiFA content could involve breeding trade-offs not apparent when selecting for FAox, FPA, and TriFA content, though this trade-off remains a hypothesis to be tested rather than a confirmed breeding constraint.

For the antioxidant capacity associated with phenolic acids, the QTL regions identified represent a particular case, as no genes putatively associated with phenolic biosynthesis were found in the corresponding bins when compared with reports in MaizeGDB. The only information related to biotic resistance associated with these bins is a gene against the fungus *Puccinia sorghi*, which infects maize in subtropical, temperate, and highland environments with high humidity (Sserumaga et al., 2020) . The lack of biosynthesis related genes in these bins is nonetheless informative; phenolic acids participate in multiple plant physiology processes related to adaptation and resistance, and their accumulation can be triggered by both mechanical and biological stress (García-Lara and Bergvinson, 2014). Changes in *p*-coumaric acid levels, for example, have been proposed to function as developmental signals (Atanasova-Penichon et al., 2012).

The proximity of bins 4.00 and 4.01 warrants a cautious interpretation of the QTL associated with FAox. Given the marker density and mapping resolution of the present biparental population, it is not possible to determine whether these adjacent signals represent two closely linked loci or a single underlying genomic region. Although both bins exceeded the significance threshold, validation in larger populations and across multiple environments will be required to confirm the stability and independence of the QTL detected at bin 4.01 before its application in marker-assisted selection. A similar consideration applies to bin 4.05, where QTL were identified for both BAox and 8-O-4′-DiFA (**Table 3**). Although this co-localization may reflect pleiotropy or closely linked loci, distinguishing between these possibilities would require additional analyses, such as multi-trait QTL analysis or fine mapping.

The absence of QTLs for total phenolic acids, in contrast to the finding of QTLs for antioxidant activity, suggest that these traits may be regulated by distinct genetic mechanisms or that the genetic architecture of total phenolic content is more complex, requiring larger populations or multiple environments for reliable QTL detection. The QTLs identified for *p*-coumaric acid on bins 1.05 and 10.02 co-localize with genes putatively involved in biotic stress resistance and phenolic biosynthesis (Ogliari et al., 2005). No common regions for single phenolic acids were found in the previous report performed on F_2:3_ families (García-Lara et al., 2010), suggesting that advancement to F_6_ allowed the discovery of novel genomic regions.

The detection of QTLs associated with diferulic and triferulic acids expands current understanding of the genetic architecture underlying these complex phenolic compounds. Ferulate residues esterified to arabinoxylans undergo oxidative coupling reactions that generate DiFA and TriFA cross-links, reinforcing the polysaccharide network and, during lignification, establishing additional linkages with lignin. Consequently, genetic variation affecting these compounds is expected to influence cell wall architecture and mechanical properties associated with plant defense (Buanafina and Morris, 2022; Chateigner-Boutin et al., 2016). The overlap of QTLs for DiFA and TriFA with genomic regions containing genes putatively associated with insect resistance and kernel hardness is therefore biologically consistent with the proposed role of these compounds in cell wall reinforcement and defense. However, recent studies indicate that the relationship between hydroxycinnamate accumulation and resistance-related phenotypes may be trait-dependent, emphasizing the need for functional validation of candidate loci before their implementation in breeding programs (López-Malvar et al., 2025). The relatively high phenotypic variation explained by the QTL detected for 8-8′, 8-O-4′-TriFA (37.48%) indicates a substantial genetic contribution to the accumulation of this compound within the evaluated population. Although this estimate was obtained from a single environment and should therefore be confirmed under additional environments, the magnitude of the effect and the biological relevance of the co-localized candidate genes support this region as a promising target for future validation. The range of phenotypic variation observed across traits in this study (2.95%–37.48%) is consistent with findings from recent QTL studies for other kernel quality traits in maize, where individual loci typically account for 2.60%–17.24% of phenotypic variation, reflecting the polygenic nature of complex grain quality traits (He et al., 2025).

For the 8-5’-DiFA trait, both bins (1.08 and 8.05) share common features, including previously reported QTLs associated with European corn borer response and 300-kernel weight (**Table 4**). Additionally, bin 1.08 contains a soft endosperm gene, while bin 8.05 includes genes associated with response to *Exserohilum turcicum.* A QTL for maysin content QTL has also been reported in this bin. Maysin is a flavone-derived compound also involved in resistance against corn earworm (Meyer et al., 2007). Interestingly, genomic regions within bin 1.08 have also been associated with resistance to Mediterranean corn borer in an independent RIL population, further supporting the biological relevance of this chromosomal region for insect resistance (Samayoa et al., 2015).

The genomic region for 5-5′-DiFA (bin 6.00) includes previously reported loci related to maize resistance (**Table 4**). One of these corresponds to a gene associated with resistance to *Bipolaris maydis*, a foliar pathogen that causes severe yield losses under warm and humid temperate and tropical conditions. These environmental conditions also favor insect infestation, suggesting that this genomic region may contribute to multiple stress-related responses. This region also co-localizes with defense-related phytochemical QTL (**Table 3**). This co-localization may reflect the metabolic relationship between phenolic acids and flavonoids, as phenolic acids are synthesized upstream of flavonoid biosynthesis (Niu et al., 2023).

Several genomic regions associated with DiFAs were also identified in a previous study performed on F_2:3_ families. Specifically, 5-5’-DiFA was consistently detected in bin 6.01 in both populations, whereas 8-5’-DiFA was associated with bins 1.08 and 8.05 in both studies, highlighting these regions as consistent QTL candidates for MAS (García-Lara et al., 2010; Ordas et al., 2010; Samayoa et al., 2015).

The QTL for 5-5’, 8-O-4(H_2_O)-TriFA on bin 7.03 overlaps with resistance-associated features documented in MaizeGDB (**Table 4**). This bin harbors a soft endosperm gene and QTLs for 300-kernel weight, sugarcane borer response, and European corn borer response, linking this TriFA isomer to a genomic region with a well-established role in physical and biotic defense. The genomic bins associated with 8-O-4’, 8-O-4’-TriFA are similarly enriched for resistance-related features. In bin 2.10, both a soft endosperm gene and a peroxidase gene (**Table 4**) are located, the latter being broadly distributed across the phytochemical traits analyzed in this study, consistent with the role of peroxidases in ferulic acid polymerization (García-Lara et al., 2007).

Given that the QTL analysis for 8-8’, 8-O-4’-TriFA was conducted in a single environment, some of the detected regions may not fully replicate across environments; however, the high phenotypic variance explained by the major QTL (37.48%) and the biological relevance of the co-localized genes strongly support their candidacy for further validation. The genomic bins associated with this compound are consistently linked to stress-related characteristics, as summarized in **Table 4**. Notably, soft endosperm genes are reported in bins 2.03, 3.07, and 3.08, a pattern that recurs across multiple phenolic acid traits in this study, suggesting a shared genetic architecture between cell wall phenolic accumulation and kernel physical defense. Bin 2.03 additionally harbors a peroxidase gene and a QTL for European corn borer response, while bins 3.07 and 3.08 share a QTL for southwestern corn borer. Class III peroxidases catalyze the oxidative coupling of ferulate residues, generating diferulate and triferulate bridges that reinforce arabinoxylan networks within grass cell walls. These cross-links increase wall rigidity, promote tissue cohesion, and contribute to resistance against insects and pathogens, making the co-localization of TriFA QTL with peroxidase genes biologically plausible (Chateigner-Boutin et al., 2016; Buanafina and Morris, 2022; López-Malvar et al., 2025). Bin 6.01 is recognized as a resistance hotspot containing two genes for response to *B. maydis*, including the major recessive gene *rhm1*, as well as QTLs for corn earworm resistance and maysin content (Balint-Kurti et al., 2008; Nisa et al., 2024). Bin 6.06 contains insect resistance genes and QTLs for response to *Cercospora zeina* and corn borer (Berger et al., 2014; López-Malvar et al., 2024).

To the best of our knowledge, this is the first study to report QTLs associated with triferulic acids in maize. TriFAs represent the most complex oxidative coupling products of ferulic acid in the cell wall, and their accumulation likely reflects the activity of class III peroxidases and laccase-type oxidases acting on pre-existing diferulic bridges. The co-localization of TriFA QTLs with peroxidase genes (bins 2.03 and 2.10) is therefore mechanistically coherent and opens a compelling avenue for functional validation (Barrière et al., 2015). Additionally, bin 2.03 harbored co-localizing QTL for 8-O-4’-DiFA and 8-8’, 8-O-4’-TriFA, which showed a significant positive phenotypic correlation (r=0.797, *p* < 0.01; **Figure 1**). This co-localization may reflect pleiotropy or close physical linkage; distinguishing between these possibilities would require further analysis. The genomic regions identified here, particularly those related to TriFAs, represent novel targets for marker-assisted selection in maize breeding programs seeking to enhance cell wall integrity and biotic stress resistance in tropical germplasm. Further studies evaluating TriFA QTLs across multiple environments and genetic backgrounds will be essential to confirm the stability of these loci and to prioritize candidates for fine mapping and gene validation. Together, the QTL regions identified in this study provide a foundation for the investigation of candidate genes regulating phenolic acid accumulation in maize kernels and represent actionable targets for marker-assisted selection in breeding programs aimed at improving cell wall quality and biotic stress resistance in maize (He et al., 2025).

In this study, the 100 most extreme RILs were selected from a population of 397 families for QTL mapping. This strategy increases the statistical power to detect loci associated with phenolic acid variation because the greater phenotypic contrast between extreme lines enhances QTL detection (Mansur et al., 1993). However, it may also lead to overestimation of the effects of some QTLs and limits the accurate estimation of population parameters such as heritability. Despite these limitations, previous studies have demonstrated the usefulness of this approach for identifying QTL associated with complex traits in maize, including insect resistance (Castro-Álvarez et al., 2015). Thus, although this approach involves methodological trade-offs, it remains an effective strategy for QTL discovery in complex traits.

## 5 Conclusion

Identifying QTLs associated with phenolic acids and antioxidant activity provides valuable targets for marker-assisted selection in maize breeding programs. The co-localization of these QTLs with genes involved in biotic stress resistance highlights their potential for developing maize varieties with enhanced pest resistance. In this study, twelve traits related to phenolic compounds were analyzed using an advanced F_6_ RIL population, revealing QTLs for nine traits, with 1 to 6 regions per trait explaining 2% to 37% of phenotypic variation. Several QTL identified in this study overlapped with genomic regions previously detected in the corresponding F_2:3_ population, particularly for 5-5′-DiFA and 8-5′-DiFA, supporting the consistency of these genomic regions in the advanced mapping population. In addition, novel QTL were identified, including the first reported loci associated with triferulic acids in maize. Functional validation of candidate genes together with evaluation of these QTL across multiple environments remain important next steps.

## Data Availability

The original contributions presented in the study are included in the article/**Supplementary Material**, further inquiries can be directed to the corresponding author.
