# Supplementary material for: QTLs associated with phenolic acid accumulation and antioxidant activity in tropical maize
Source: Front Plant Sci. 2026 Aug 18;17:1876404. doi: 10.3389/fpls.2026.1876404 (PMC13529940; doi:10.3389/fpls.2026.1876404)
Supplement: Supplementary file 1 [file Table1.docx]

**Supplementary Material**

**Supplementary Figures**

**Figure S1.** Violin plots showing the distribution of P84 (red), Kilima (blue), and 100 F₆ RIL families (green) for A) total free phenolics, B) total bound phenolics, C) free antioxidant activity, and D) bound antioxidant activity. The x-axis shows genotype category. The y-axis shows trait values (mg GAE/100 g dw for phenolics; µmol Trolox/100 g dw for antioxidant activity). Points represent individual values within each genotype group.
